# Enhanced Stability and Bioactivity of Natural Anticancer Topoisomerase I Inhibitors through Cyclodextrin Complexation

**DOI:** 10.3390/pharmaceutics13101609

**Published:** 2021-10-03

**Authors:** Víctor González-Ruiz, Ángel Cores, Olmo Martín-Cámara, Karen Orellana, Víctor Cervera-Carrascón, Patrycja Michalska, Ana I. Olives, Rafael León, M. Antonia Martín, J. Carlos Menéndez

**Affiliations:** 1 Swiss Centre for Applied Human Toxicology, School of Pharmaceutical Sciences and Institute of Pharmaceutical Sciences of Western Switzerland, University of Geneva, 1205 Geneva, Switzerland; victor.gonzalez@unige.ch; 2 Unidad de Química Orgánica y Farmacéutica, Departamento de Química en Ciencias Farmacéuticas, Facultad de Farmacia, Universidad Complutense de Madrid, 28040 Madrid, Spain; acores@ucm.es (Á.C.); olmomart@ucm.es (O.M.-C.); 3 Facultad de Química y Farmacia, Universidad Nacional Autónoma de Honduras (UNAH), 11101 Tegucigalpa, Honduras; korellana@unah.edu.hn; 4 Unidad de Química Analítica, Departamento de Química en Ciencias Farmacéuticas, Facultad de Farmacia, Universidad Complutense de Madrid, 28040 Madrid, Spain; vcervera@ucm.es (V.C.-C.); aiolives@ucm.es (A.I.O.); 5 Instituto Teófilo Hernando y Departamento de Farmacologia y Terapéutica, Facultad de Medicina, Universidad Autónoma de Madrid, 28049 Madrid, Spain; patrycjamdziama@gmail.com; 6 Instituto de Química Médica, Consejo Superior de Investigaciones Científicas, 28006 Madrid, Spain; rafael.leon@iqm.csic.es

**Keywords:** molecular recognition, cavitands, supramolecular complexes, anticancer agents, drug stabilization

## Abstract

The use of cyclodextrins as drug nano-carrier systems for drug delivery is gaining importance in the pharmaceutical industry due to the interesting pharmacokinetic properties of the resulting inclusion complexes. In the present work, complexes of the anti-cancer alkaloids camptothecin and luotonin A have been prepared with β-cyclodextrin and hydroxypropyl-β-cyclodextrin. These cyclodextrin complexes were characterized by nuclear magnetic resonance spectroscopy (NMR). The variations in the ^1^H-NMR and ^13^C-NMR chemical shifts allowed to establish the inclusion modes of the compounds into the cyclodextrin cavities, which were supported by docking and molecular dynamics studies. The efficiency of the complexation was quantified by UV-Vis spectrophotometry and spectrofluorimetry, which showed that the protonation equilibria of camptothecin and luotonin A were drastically hampered upon formation of the inclusion complexes. The stabilization of camptothecin towards hydrolysis inside the cyclodextrin cavity was verified by the quantitation of the active lactone form by reverse phase liquid chromatography fluorimetric detection, both in basic conditions and in the presence of serum albumin. The antitumor activity of luotonin A and camptothecin complexes were studied in several cancer cell lines (breast, lung, hepatic carcinoma, ovarian carcinoma and human neuroblastoma) and an enhanced activity was found compared to the free alkaloids, particularly in the case of hydroxypropyl-β-cyclodextrin derivatives. This result shows that the cyclodextrin inclusion strategy has much potential towards reaching the goal of employing luotonin A or its analogues as stable analogues of camptothecin.

## 1. Introduction

Topoisomerase I is a very relevant, although relatively unexploited, anticancer target [1,2]. Camptothecin (CPT), an alkaloid from *Camptotheca acuminata*, was the first anti-cancer compound that was proved to exert topoisomerase I inhibition. This mechanism raised much interest, but the clinical application of camptothecin was hampered by its poor water solubility and its low chemical stability, which is due to the very fast hydrolysis of the essential lactone ring to the corresponding hydroxy acid [3]. Besides leading to a lower anticancer activity, this ring-opening process is associated to an increased renal toxicity [4,5,6]. The potent anticancer activity of camptothecin, together with its novel mechanism of action, prompted efforts towards improving its stability and bioavailability. Several delivery systems have been explored for this purpose, and thus copolymers of poly(ethylene glycol) and poly(γ-benzyl L-glutamate) have been shown to form stable micelles in aqueous solutions containing camptothecin [7] and chitosan-folate has been conjugated to poly(vinyl alcohol) microcapsules able to load camptothecin [8]. Nanoparticles based on silica covalently bound to camptothecin reduce its systemic toxicity while maintaining its cytotoxic activity [9], although the significant toxicity of nanoparticles by themselves needs to be considered [10].

**Figure 1:**
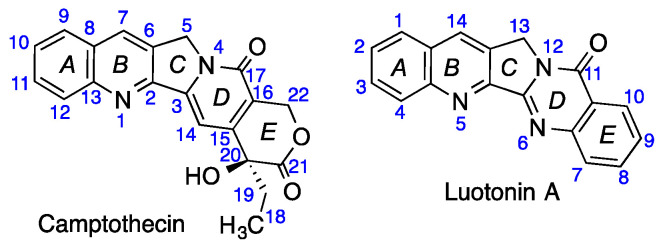
Structures and numbering of camptothecin and luotonin A.

Luotonin A (Figure 1) is a natural analogue of camptothecin that also inhibits topoisomerase I and has the advantage of being chemically stable. Unfortunately, it also shows a lower activity than camptothecin, a limitation that is at least partly associated to its very low solubility. Its good stability profile has stimulated the synthesis and study of many analogues of the natural product for their study as potential anticancer agents [11,12,13,14,15,16] and, more recently, as agrochemicals due to their antifungal, antiviral and insecticidal properties [17,18]. The alternative approach involving the development of new forms of administration of the natural product that provide an improvement of its bioavailability is still unexplored.

A suitable stability and solubility in water are essential properties for a drug or drug candidate, as they are closely related to bioavailability. Many anti-cancer drugs suffer from low aqueous solubilities and this problem prevents their oral administration [19]. This challenge can be met by the formulation of these drugs in nano-delivery water-soluble systems [20,21]. In particular, inclusion of drugs into cyclodextrin (CD) cavities is well-known to not only alleviate problems related to their low solubility [22], but also to increase their chemical stability [23] and, often, enhance their pharmacological properties [24]. To cite some recent examples, CDs favor the intestinal absorption of curcumin [25], decrease the degradation rate of benzylpenicillin [26], allow the intraoral release of fluconazole [27] and albendazol [28] and increase the solubility and stability of the multitarget neuroprotective agent ITH-12674 [29]. The pharmacokinetic profile of the potential anticancer agent phenoxodiol was notably improved through β-cyclodextrin complex formation, thereby leading to reduced side effects [30]. Cyclodextrins are generally recognized as safe both orally and parenterally and are indeed widely employed in the pharmaceutical industry and as food additives [31].

The formulation of drugs as supramolecular complexes with cyclodextrins is well known and examples of such complexes are described in several pharmacopoeias. They often solve stability and solubility issues while showing diminished toxicity and negative side effects than other alternatives. Because of its high anticancer activity, hampered by its low solubility and stability, camptothecin is an excellent candidate for studying the benefits derived from complexation with CDs. The interaction of CPT and its derivative SN-38 with CDs has been studied in order to enhance fluorimetric detection of both analytes in human urine [32]. The affinity constants of CPT-CD inclusion complexes have been determined based on the variations of high-performance liquid chromatography (HPLC) retention indexes [33], and also on the changes of the apparent solubility of the guest molecules in the presence of α-, β- and γ-cyclodextrin [34]. The effect of hydroxypropyl-β-cyclodextrin (HP-β-CD) complexation and pH on the solubility properties of camptothecin have also been reported [35]. The binding stoichiometry, binding constants, and inclusion modes of some water-soluble, negatively charged cyclodextrin derivatives with irinotecan, topotecan, and doxorubicin as guest molecules have been determined in solution through drug titration with increasing amounts of the CDs [36]. Regarding the biological activity of CPT-CD complexes, an in vitro cytotoxicity assay of the complexes of 9-nitro CPT with HP-β-CD was carried out in several tumor cell lines, namely S 180, Skov-3, Michigan Cancer Foundation-7 (MCF-7) and HeLa. A slight improvement in the anti-tumor activity was observed, which the authors attributed to the higher stability of the active principle inside the complexes [37,38]. Finally, modified cyclodextrin complexes containing camptothecin were shown to improve its bioavailability [39,40]. Luotonin A is much less studied in this regard, although solubility assays have been carried out in the presence of β-CD and HP-β-CD [41]. In the precedent examples, the camptothecin and luotonin A CD complexes were prepared and studied in solution, and no effort was made to isolate and characterize them in purified form.

In this context, we describe herein the experimental conditions for synthesizing and isolating inclusion complexes of camptothecin and the related alkaloid luotonin A with β-cyclodextrin and hydroxypropyl-β-cyclodextrin. The solid complexes were first characterized by ^1^H-NMR and ^13^C-NMR, and the efficiency of the complexation was verified by additional spectroscopic techniques (UV-Vis absorption and fluorescence emission). An increase in camptothecin stability due to its inclusion in the CD nanocavities was shown through the quantitation of lactone and carboxylate forms by HPLC. The CD complexes showed a noticeably increased cytotoxic activity against breast, lung, liver, ovarian and neuroblastoma cell lines.

## 2. Materials and Methods

### 2.1. Instruments and Materials

Ultraviolet-visible (UV-Vis) absorption spectra were recorded with an Agilent Cary 60 (Santa Clara, CA, USA) equipped with the software Cary WinUV. Corrected excitation and emission spectra and measurements at fixed wavelengths were obtained with a Horiba-Jobin Yvon (Edison, NJ, USA) FluoroMax-4P spectrofluorometer equipped with the control and data acquisition software FluorEssence 2.1. ^1^H-NMR and ^13^C-NMR spectra were recorded in d_6_-DMSO with a Bruker Avance III 700 MHz spectrometer (Billerica, MA, USA), maintained by the Servicio de Resonancia Magnética Nuclear, UCM; chemical shifts were referred to the residual solvent signal.

The chromatographic study of camptothecin stability was carried out with a liquid chromatographic system (Merck-Hitachi, Tokyo, Japan) consisting of a quaternary gradient pump L-7100, able to handle a maximum backpressure of 412 bar, and a fluorescence detector L-7485 and a L-2300 oven. Data was acquired through a PC running HPLC System Manager software, version 4.1 (Merk-Hitachi). The samples were injected through a Rheodyne 7725i injector.

A rotatory shaker (Stuart Rotator SB3, Stone, Staffordshire, UK) and an ultrasonic bath (Elmasonic S15, Singen, Germany) were employed for the preparation of the inclusion complexes by mixing and stirring the alkaloids and CDs. A Crison micro-pH 2001 pH-meter (Barcelona, Spain) was used to monitor the final pH values of the buffer solutions.

Commercially available camptothecin, luotonin A, as well as β-cyclodextrin (β-CD) and (hydroxypropyl)-β-cyclodextrin (HP-β-CD) with a 1.0 molar substitution pattern were purchased from Sigma-Aldrich (Steinheim, Germany). All reagents and solvents were spectroscopic or chromatographic grade and were used without further purification. Ultrapure water was obtained from a Milli-Q Direct 8 system (Millipore, Molsheim, France). d_6_-DMSO and D_2_O were employed as solvents for obtaining ^1^H-NMR and ^13^C-NMR spectra.

### 2.2. Complexation Studies in Solution

A stock solution (1.0 × 10^−3^ M) of camptothecin or luotonin A in DMSO was prepared. Adequate aliquots of this solution were taken to prepare a 1.0 × 10^−5^ M solution in ethanol and their UV-Vis spectra were obtained in order to determine the concentration of the alkaloids in this solution, using literature values [42,43] for their molar absorptivities. Twelve aqueous cyclodextrin solutions ranging from 1.0 × 10^−4^ to 1.0 × 10^−2^ M at equal concentration intervals were prepared. An aliquot of 5 mL of each of these solutions was added to 50 μL of the 1.0 × 10^−5^ M camptothecin or luotonin A ethanolic solution, to reach a final alkaloid concentration of 1.0 × 10^−7^ M. Then, 100 μL of 5.0 M aqueous HCl were added to provide a final pH value of 1.0. The same procedure was employed for preparing the complexes in aqueous solution (pH 5.5). Solutions containing the alkaloids in acid aqueous solution but lacking the cyclodextrin were also prepared, to be used as references. All complex solutions were prepared in duplicate sets.

These solutions were magnetically stirred at room temperature for 2 h and their fluorescence spectra were recorded. Additional measurements were performed after 6, 24 and 48 h.

### 2.3. Synthesis and Isolation of Inclusion Complexes and Their NMR Characterization

The inclusion complexes were prepared from an accurately weighed amount of the suitable anticancer drug (camptothecin or luotonin A) and cyclodextrin to reach a 1:1 guest:host molar ratio, then an adequate volume of deionized and bi-distilled water was added to produce a final alkaloid cyclodextrin concentration of 1.0 × 10^−2^ M in water. The aqueous suspensions thus obtained were maintained under sonication in an ultrasound bath for 30 min. Next, the drug-CDs mixtures were magnetically stirred (1250 rpm) for 23.5 h. These steps (sonication for 30 min followed by magnetic stirring during 23.5 h) were repeated through five days. The suspensions obtained were stored at 4 °C for five days and centrifuged at 5000 rpm to separate the solid pellet from the supernatants. Finally, both the solids and the supernatants were freeze-dried during 48 h. The loads of ligands in the complexes were calculated from their UV-Vis absorption spectra, considering the molar absorptivity values of the alkaloids [42,43], and were around 50%.

The effective inclusion of the drug molecules into the cyclodextrin cavities was verified by quantifying the changes in the chemical shifts of the signals of the molecules in the inclusion complexes in comparison to those obtained for the free alkaloids under the same experimental conditions. For this purpose, an amount of the complexes containing 1–2 mg of one of the alkaloids were accurately weighed and dissolved in deuterated water (D_2_O) or hexadeuterated dimethylsulfoxide (d_6_-DMSO) and the ^1^H-NMR and ^13^C-NMR spectra were registered.

### 2.4. Computational Study of the Alkaloid-CD Complexes

Docking studies were carried out using AutoDock Vina (The Scripps Research Institute, La Jolla, CA, USA) to predict the configuration of the inclusion complex between β-CD and HP-β-CD with ligands. The structure of β-CD was obtained from Protein Data Bank (PDB ID: 2Y4S) while HP-β-CD was built by adding hydroxypropyl side chains to the C-6 position of β-CD, and both structures were optimized with the MM2 force field using ChemBio3D Ultra 12.0 (CambridgeSoft, Cambridge, MA, USA). Luotonin A and camptothecin were drawn in ChemBioDraw Ultra 12.0 (CambridgeSoft) and optimized by the MM2 method in ChemBio3D Ultra 12.0. The non-polar hydrogen atoms were merged, and Gasteiger charges were added for all atoms of optimized molecules using AutoDockTools. The ligands were separately docked into this grid with the genetic algorithm (GA) as implemented in AutoDock Vina. The resulting docking mode was visualized using PyMol and the best results were chosen to perform molecular dynamics simulations.

Molecular dynamics (MD) simulations were performed using Gromacs 2018.1. The CHARMM36 force field was employed to study the complex formation. The topologies were created with CgenFF. The complex was solvated using SPC water model, and then minimized. A two-stage equilibration was done by applying NVT ensemble followed by NPT ensemble for 50,000 steps of 2 fs each. A 50 ns simulation was performed for each system, with a time step of 2 ps and a cut-off of 1.0 nm. The long-range electrostatic energies were calculated with PME method, with a four order cubic interpolation and a spaced grid of 0.16 nm. The temperature was regulated at 300 K using a Berendsen thermostat with a coupling constant of 0.1 ps. The pressure was fixed at 1 bar and control with a Parrinello-Rahman barostat with a coupling constant of 2 ps and a compressibility of 4.5 × 10^−5^ bar^−1^was employed. Root-mean-square displacement (RMSD) between any snapshot and the minimized state of the system was calculated to evaluate the equilibrium of the system during simulation. The mobility of the ligand inside the CD cavity was evaluated by measuring distances between the center of mass of the CD and different points of the different ligands. An estimation of the binding energy was also calculated by using the molecular mechanics Poisson-Boltzman surface area (MM-PBSA) method using the last 1000 MD snapshots (10 ns) from every simulation.

### 2.5. Verification of the Formation of Inclusion Complexes by UV-Vis Absorption and Fluorescence Spectrometries

Complexation efficiency was verified through titration of aqueous solutions of inclusion complexes CPT/HP-β-CD and CPT/β-CD with hydrochloric acid (10 M). The differences in the UV-Vis absorption spectra and the titration curves of camptothecin (1.0 × 10^−5^ M) and its inclusion complexes are described below. Similarly, to verify the efficiency of complexation for luotonin A (1.0 × 10^−6^ M), titrations with perchloric acid (1.0 M) of free alkaloid and the corresponding inclusion complexes were also carried out. Emission spectra were recorded for λ_ex_ = 342 nm (neutral form) and λ_ex_ = 392 nm (cationic form). The changes in the fluorescence intensity allow evaluating the efficiency of the protection afforded by CDs to the alkaloids.

Solutions of the free alkaloids for the reference acid titration experiments were prepared at 1.0 × 10^−3^ M in DMSO to assure complete dissolution. Then, aqueous solutions were prepared at 1.0 × 10^−5^ M for UV-Vis or 1.0 × 10^−6^ M for fluorescence experiments.

### 2.6. HPLC-FL Evaluation of the Stability of Camptothecin Inclusion Complexes

The determination of lactone and carboxylate forms of camptothecin as free drug as well as the corresponding inclusion complexes was tracked by HPLC with fluorescence detection. A Luna C-18 (2) column (150 × 3.0 mm packed with 5.0 μm of particle size), manufactured by Phenomenex (Torrance, CA, USA), was employed. Mobile phases were ultrasonically degassed prior to their use and were then filtered under low-pressure through Polyamid 47 mm filters (Sartolon 0.45 μM pore size, Sartorius Stedim Biotech, Goettingen, Germany). Chromatographic separations were developed under isocratic conditions using a mobile phase composition of acetonitrile:water 40:60; v:v as the mobile phase at a flow rate of 0.5 mL min^−1^ and at 25 °C. The fluorescence detection conditions were λ_ex_ = 370 nm, λ_em_ = 440 nm. camptothecin and luotonin A solutions were prepared from accurately weighed amounts of the solid products, dissolved in dimethylsulfoxide (DMSO) at 1.0 × 10^−3^ M concentration. The concentration of camptothecin and luotonin A were verified by UV-Vis spectrophotometry taking into account their molar absorptivities [42,43]. Standard solutions of luotonin A and camptothecin were chromatographed independently to establish the retention time and in order to obtain the calibration curves in the range of 1.0 × 10^−10^ M to 5.0 × 10^−8^ M for quantitative analysis. Due to its better chemical stability, luotonin A was employed as internal standard (IS) to calculate camptothecin concentrations. The concentration of luotonin A was kept constant in all solutions at 1.0 × 10^−9^ M while the camptothecin concentration varied. The solutions of the standards were prepared from the stock solution in DMSO (1.0 × 10^−3^ M) and were then diluted with acetonitrile kept in an ice bath. The final solutions to be injected in the chromatographic system were prepared in acetonitrile:water (40:60 v:v) or in acetonitrile:acetic acid/ammonium acetate buffered aqueous solution (0.15 M) at pH 3.0; 6.0 and 8.0 (40:60; v:v). Calibration curves were obtained by plotting the camptothecin/luotonin A area ratio of the chromatographic peaks vs. the theoretical concentrations of camptothecin.

For the chromatographic analysis of the inclusion complexes, solutions of the solid CPT/HP-β-CD and CPT/β-CD inclusion complexes were dissolved in DMSO and then diluted in mobile phase to reach a final concentration around 10^−5^ M. The concentration of camptothecin in the complexes was verified by UV-Vis spectrometry as described above. The solutions containing the CPT/CDs complexes were diluted in MeCN kept in an ice bath and then an adequate volume of luotonin A (IS) was added to produce the final concentration of camptothecin in the complexes (5.0 × 10^−9^ M) and the IS (1.0 × 10^−9^ M). The solutions to be injected in the HPLC instrument contained the complexes with the internal standard dissolved in the acetonitrile:water (40:60 v:v) mobile phase, or in MeCN: buffered acetonitrile:buffered aqueous acetic acid/ammonium acetate 0.15 M at pH 6.0 (40:60; v:v). All the sample/standard solutions were filtered through 4 mm syringe filters (Phenex, Nylon, 0.45 μm pore size, Phenomenex, Torrance, CA, USA) prior to their injection.

The lactone-carboxylate equilibrium was studied by daily preparing fresh solutions of CPT/CD and using luotonin A as an internal standard. These solutions were maintained in an ice bath and immediately chromatographed. Then, 10 µL of aqueous ammonia was added to produce a 0.15 M concentration of NH_3_ in the solutions of the CPT and the CPT/CDs complexes, and these alkalinized solutions were injected in the HPLC instrument immediately (t = 0) and after 15 min at ambient temperature.

### 2.7. Cell Lines Culture

Cells were harvested in a 75 cm^3^ flask and incubated at 37 °C with a wet atmosphere containing 5% of CO_2_. Cells were maintained using the following media: (a) SH-SY5Y cells: MEM/F12 containing 15 non-essential amino acids and supplemented with 10% of fetal bovine serum (FBS), glutamine 1 mM, 50 units/mL of penicillin and 50 μg/mL of streptomycin (GIBCO, Madrid, Spain). (b) AREc32 cells (kindly shared by Dr. C. R. Wolf): DMEM with glutamax, supplemented with 10% fetal bovine serum (FBS), 1% of the penicillin-streptomycin antibiotic combination, and geneticin (0.8 mg/mL, G418) (GIBCO, Madrid, Spain). (c) HepG2 cells: DMEM supplemented with 10% FBS, 1% of the antibiotics penicillin and streptomycin. (d) H23 and A2780 cell lines: RPMI-1640 supplemented with 10% fetal bovine serum (FBS), 1% of the antibiotics penicillin and streptomycin.

### 2.8. Cytotoxic Effects of the Alkaloid-CD Complexes

Cells were seeded in 96-well plates at a density of SH-SY5Y: AREc32 and HepG2: 30,000 cells/well; H23 and A2780: 10,000 cells/well, and maintained during 24 h. Thereafter, cells were treated with the desired complex or compound at increasing concentrations (camptothecin and its complexes: 0.01, 0.1, 1 and 10 µM; luotonin and its complexes: 0.1, 1, 10, 30 and 60 µM) during 72 h including camptothecin and luotonin A as control and reference compounds. To this end, stock solutions (10 mM) of complexes and reference compounds were prepared in DMSO and kept at −20 °C. Final serial solutions were prepared in the correspondent cell line culture media. Finally, cell viability was addressed by the MTT reduction method. MTT (5 mg/mL, 10 µL/well) was added and incubated for 2 h. Then, the medium was removed and the purple formazan crystals formed by viable cells were dissolved using 100 µL of DMSO. Finally, absorbance was measured at 535 nm in a microplate reader (SPECTROstar NANO, BMG Labtech). Basal absorbance was set to 100% and results were normalized to basal condition. The cytotoxic concentration to obtain 50% viability reduction (CC_50_) for camptothecin, luotonin A and complexes were calculated from dose-response curves represented as percentage of cell death vs. concentration of alkaloid or complex, fitted by non-linear regression and data interpolated to value 50.

## 3. Results and Discussion

### 3.1. Studies of Supramolecular Complex Formation in Solution

We started our work by examining the changes in the fluorescence spectra of the alkaloids in the presence of increasing amounts of both cyclodextrins under study, as a proof of their inclusion. Both alkaloids have at least one basic nitrogen that can be protonated under strongly acidic conditions, and it can be reasonably assumed that the more lipophilic neutral form is the one that is primarily included in the cyclodextrin cavity. In the case of camptothecin, the fluorescence emission of the cationic form has a maximum at 510 nm, while the corresponding neutral form emits at 428 nm [42,44]. The corresponding values for luotonin A are λ_em_ (cationic form) = 502 nm and λ_em_ (neutral form) = 426 nm [43].

**Figure 2:**
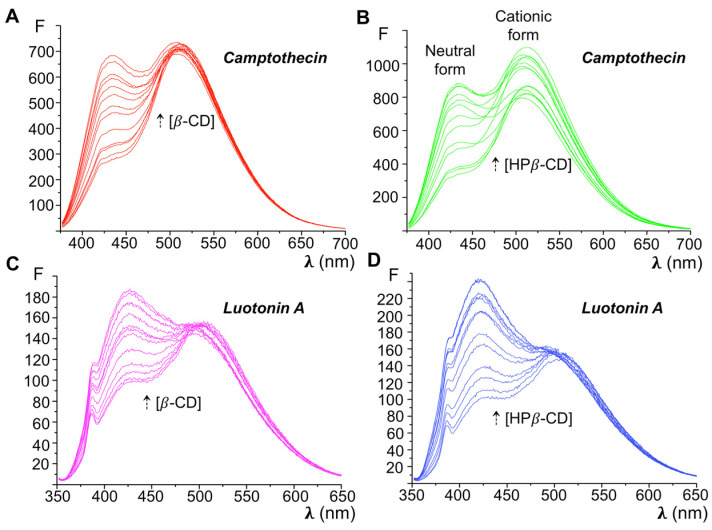
Initial studies that prove the formation in solution of inclusion complexes of camptothecin and luotonin A with β-CD and HP-β-CD. Fluorescence emission spectra of camptothecin in the presence of: (**A**) increasing β-CD concentrations; (**B**) increasing HP-β-CD concentrations. Fluorescence emission spectra of luotonin A in the presence of: (**C**) increasing β-CD concentrations; (**D**) increasing HP-β-CD concentrations. The complexes were prepared in acidic media.

Figure 2A shows the fluorescence emission spectra of camptothecin at pH = 1 in the presence of increasing amounts of β-cyclodextrin. The blank solution lacking cyclodextrin clearly shows the absorption maximum at 510 nm that is characteristic of the cationic form, and only a shoulder at 428 nm, corresponding to the neutral form. The solutions containing β-CD show increasing fluorescence intensities for the neutral form, which becomes a well-define peak starting from 10^−3^ M CD concentration. The appearance of the neutral camptothecin form, even under the highly acidic conditions, is a proof of inclusion of camptothecin in the CD cavity and underlines its ability to solubilize the neutral form of the alkaloid. The same behavior was observed for the camptothecin-HP-β-CD, luotonin-β-CD and luotonin HP-β-CD combinations (Figure 2B–D, respectively).

The changes observed in the fluorescence intensity can be exploited for the quantitative evaluation of the association constants (*K*_ass_), which are related to the stability of the complexes [45,46]. Thus, fluorescence intensities obtained for maxima corresponding to the neutral form of camptothecin and luotonin A (Supplementary Materials Figures S1–S4) were used for calculating *K*_ass_ according to the Benesi–Hildebrand model for two pH values (Table 1). In the case of camptothecin, the association constant values obtained are in agreement with those described in the literature, even though previously *K*_ass_ values were deduced from solubility experiments [34,35,41]. The association constants of the luotonin A complexes were in the same range of those of camptothecin, as expected for a compound with similar chemical structure. The association constants in acidic media (pH = 1.0) are lower than in aqueous solution because the cationic form of the alkaloids is not readily included in the CD cavity and therefore tht measured fluorescence intensity values correspond to the neutral. The HP-β-CD complexes are slightly more stable than those derived from β-CD, in agreement with the results obtained for the solid complexes described below. The values summarized in Table 1 are similar other association constants of drug-cyclodextrin complexes described in the literature; for instance, the complexes of β-CD with 9-nitrocamptothecin, doxorubicin and progesterone acetate were 185, 243 (at pH = 5.90) and 243, respectively [23].

### 3.2. NMR Characterization of the Cyclodextrin Complexes

NMR spectrometry is the most powerful technique to evidence the inclusion of guest molecules into CD cavities. The proximity between groups and the interactions among the hydroxyl groups of CDs and the guest molecules causes changes in the chemical shifts as well as in the shape of the signals. These changes are more intense in the parts of the molecule that are inside the CD cavity and thus the mode of inclusion of the guest molecules into the host can be established.

**Figure 3:**
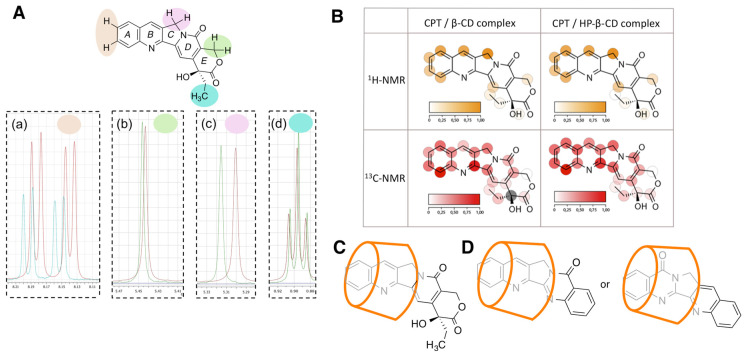
(**A**) Changes observed in selected signals of the camptothecin ^1^H-NMR spectrum upon complexation with **β**-cyclodextrin, showing significant changes in the position of the signals in (**a**) and (**c**) and only slight changes in (**b**) and (**d**). (**B**) Normalized (0–1) chemical shift changes in the ^1^H-NMR and ^13^C-NMR spectra of camptothecin following complexation with **β**-cyclodextrin and hydroxypropyl-**β**-cyclodextrin. (**C**) Model of inclusion of camptothecin inside the **β**-CD cavity according to NMR data. (**D**) Two alternative models of inclusion of luotonin A inside the **β**-CD cavity according to NMR data.

^1^H-NMR reference spectra of free camptothecin and the CPT/β-CD complex are shown in Figures S5 and S6 and the numerical data are summarized in Table S1. As a representative example of the changes observed, Figure 3A shows expansions of key regions of the superimposed ^1^H-NMR spectra of free camptothecin and the CPT/β-CD inclusion complex. The C-18 methyl group of camptothecin (triplet in box d) and the E-ring methylene (singlet in box b) show very small changes in the complex in comparison to the free drug. More marked displacements are observed for the signal of the C-ring methylene (singlet in box c) and the H-10 and H-11 aromatic protons (two doublets in box a). A similar behavior was observed in the ^1^H-NMR of the CPT/HP-β-CD inclusion complex (Figure S7 and Table S1). It is relevant to note that these data come from the freeze-dried precipitated complexes (obtained as described in Section 2.3). On the other hand, spectral changes obtained for the freeze-dried supernatants were negligible, showing the that precipitation of the complex was essentially quantitative.

The ^13^C-NMR spectra of the CPT-CD complexes show that the signal in the region at 130 ppm corresponding to the carbon on positions C-12 of camptothecin is shifted as a consequence of the inclusion into the CDs. The changes observed in the chemical shift values (^13^C-NMR spectra) with regard to those observed for the inclusion complexes are summarized in Table S2. The magnitude of the changes in the chemical shifts are visually represented in Figure 3B as shades of red or orange. As can be appreciated, the more intense changes are associated to the signals corresponding to the quinoline ring, the more lipophilic part of the pentacyclic backbone of the alkaloid. Therefore, the NMR data support the inclusion mode represented in Figure 3C, with the quinoline fragment of camptothecin placed inside the CD cavity.

In the case of the CD complexes with luotonin A, ^1^H-NMR spectra show changes in the chemical shifts of the signals around 8 ppm for both β-CD (Figure S8) and HP-β-CD (Figure S9), but the chemical shifts are generally less affected by complexation than in the case of camptothecin, as summarized in Table S3. The fact that both ends of the molecule are similarly affected by complexation suggests that two modes of inclusion are similarly stable (Figure 3D). Alternatively, these observations could be explained by the formation of a 2:1 CD-ligand complex, but this possibility was discarded computationally (see below).

### 3.3. Computational Study of the Alkaloid-Cyclodextrin Complexes

**Figure 4:**
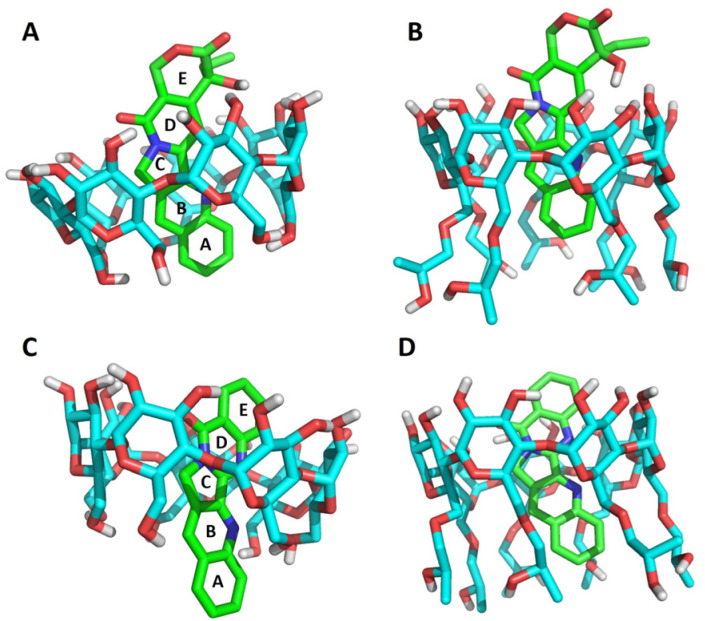
Docking of camptothecin and luotonin A inside the cyclodextrin cavities: (**A**) camptothecin–β-CD complex; (**B**) camptothecin-HP-β-CD complex; (**C**) luotonin A–β-CD complex; (**D**) luotonin A-HP-β-CD complex.

Docking studies showed that camptothecin forms complexes with both CDs having the A and B rings fully inserted in the cavity, the C ring placed in an intermediate state and the rest of the molecule outside. In the case of the luotonin A-CD 1:1 complexes, the D and E rings are completely inside the cavity, C is placed in an intermediate state and B and A are outside (β-CD complex) or resting along the hydroxypropyl chains (HP-β-CD complex) (Figure 4). An alternative pose with the A and B rings inside the cavity and the D and E rings outside showed an almost identical calculated stability, suggesting that both poses are viable and in agreement with the NMR data summarized above.

**Figure 5:**
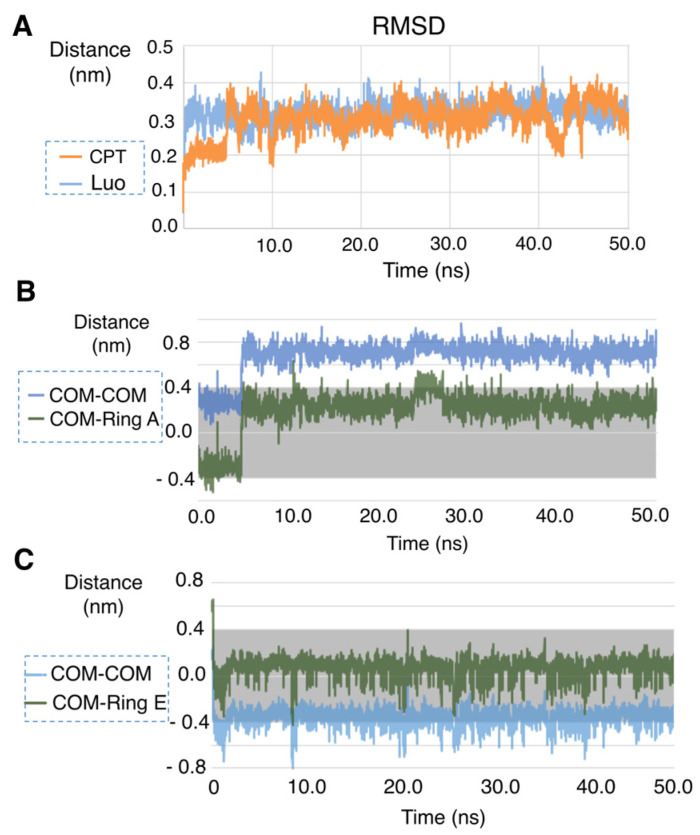
Molecular dynamics studies: (**A**) RMSD for the camptothecin-β-CD and luotonin A-β-CD complexes; (**B**) evolution of the distance between the center of mass (COM) of β-CD and the center of mass of camptothecin, and similar study of the center of ring A; (**C**) evolution of the distance between the center of mass of β-CD and the center of mass of luotonin A, and similar study of the center of ring E.

The stability of the complexes was further studied by molecular dynamics, using as example the case of β-CD. To this end, a calculation of the RMSD for the camptothecin-β-CD and luotonin A-β-CD complexes was performed by aligning every structure from the trajectory with the structure obtained after the minimization of the system. Both complexes showed good stability, and fluctuations did not exceed 4 Å. The camptothecin-β-CD complex required some time (ca. 4.8 ns) to become stabilized while the luotonin A-β-CD complex showed a stable behavior almost immediately (around 0.25 ns) (Figure 5A). On the other hand, when a hypothetical complex between luotonin A and β-CD in 1:2 stoichiometry was studied, a large fluctuation was observed (Figure S10).

Additional calculations of distances between ligands and CDs were also performed by measuring the distance between the center of mass of the CD and the center of mass of the ligand (d [COM(CD)—COM(lig)]), as well as the distance between the center of mass of the CD and the center of either ring A (for camptothecin) or ring E (for luotonin A), according to their starting binding poses. For this study, the height of the β-CD inverted cone was taken to be 7.9 Å [47]. Therefore, the center of the β-CD was placed at 3.95 Å, and this point was regarded as the zero of the measurement.

In the case of camptothecin, an evolution of the system can be appreciated, which is stabilized after ca. 4.8 ns in a slightly outer position with respect to the original docking pose. The quantitative results show that, after stabilization, the compound is inside the CD cavity the 100% of the total number of snapshots, with an average distance of 0.671 ± 0.127 nm. The behavior of the ring A of the camptothecin also shows that the compound is stabilized in a good pose (an average distance of 0.191 ± 0.174 nm), staying inside the CD cavity most of the time (94.8% of the total snapshots), as shown in Figure 5B.

The luotonin A-β-CD complex is very stable (Figure 5C). The center of mass of the alkaloid is mostly (72.4% of the snapshots) inside the CD cavity (average distance of −0.360 ± 0.080 nm), and its ring E stays really close to this center (average distance of 0.071 ± 0.084 nm) during all the simulation (99.8% of the snapshots).

### 3.4. Study of the Inclusion Complexes by UV-VIS Absorption and Fluorescence Emission

The UV-Vis absorption spectrum of camptothecin in aqueous solution (pH = 6.0) shows a band in the 310–400 nm region, which is resolved into two peaks at 353.5 and 368 nm [42]. Upon acidification of the aqueous solvent from pH 6 to 2, the absorbance of this band decreases as a consequence of the protonation of the quinoline nitrogen and a new band appears with a maximum at 400–410 nm. The formation of inclusion complexes with CDs should protect the camptothecin molecule and therefore hamper its protonation.

**Figure 6:**
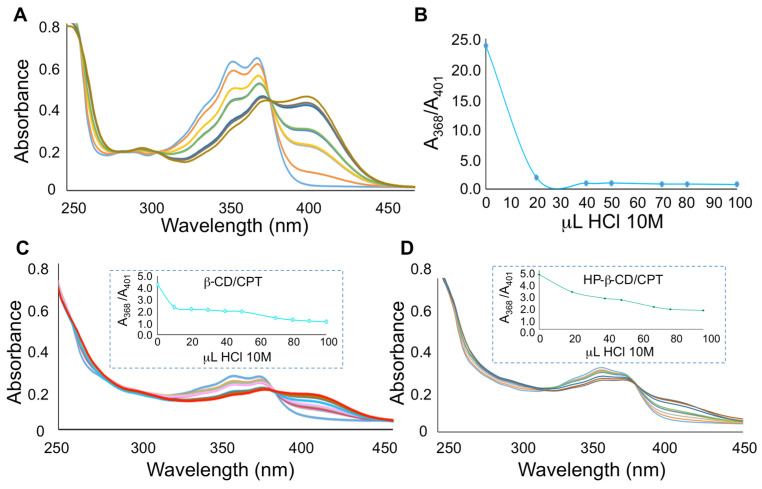
(**A**) Effect of the addition of increasing volumes (10 μL per addition) of 10 M HCl on the UV-Vis absorption spectra of camptothecin in aqueous solution. (**B**) Ratiometric titration of camptothecin. λ = 366 nm corresponds to the neutral form and λ = 401 nm corresponds to the protonated form. (**C**) Effect of the addition of increasing volumes (10 μL per addition) of 10M HCl on the UV-Vis absorption spectra of the camptothecin-β-CD complex in aqueous solution. Inset: Ratiometric titration of the camptothecin-β-CD complex. (**D**) Effect of the addition of increasing volumes (10 μL per addition) of 10M HCl on the UV-Vis absorption spectra of the camptothecin-HP-β-CD complex in aqueous solution. Inset: Ratiometric titration of the camptothecin-HP-β-CD complex.

In order to use this approach to prove the formation of inclusion complexes, solutions of camptothecin were titrated with aqueous HCl and the effect of CD complexation on these protonation equilibria were assessed. Figure 6 shows the UV-Vis absorption spectra corresponding to the titration of free camptothecin and CPT/CDs complexes with HCl solution.

Solutions of native camptothecin as well as its inclusion complexes were prepared with the amount of alkaloid suitable to achieve a 1.0 × 10^−5^ M concentration in the aqueous solution. Aqueous HCl standardized solutions with 0.1 M, 1.0 M and 10.0 M concentrations were initially assayed as titrating agents; however, we found that the lower-concentration solutions required high volumes to observe the spectral changes due to the protonation of camptothecin, and for this reason 10.0 M HCl was chosen for subsequent work. Figure 6A shows the protonation of camptothecin, revealed by the appearance of a band at 410 nm with an intensity proportional to the acid concentration and a concomitant decrease in the absorbance values at the wavelengths characteristic of the neutral form (350 and 368 nm). On the other hand, in the case of the HP-β-CD and β-CD complexes the same HCl concentration causes a weak shoulder in the 400–430 nm region and a very slight decrease in absorbance maxima corresponding to the neutral camptothecin. Therefore, the protonation of free camptothecin is easier in comparison to the CD complexes, as the accessibility of CPT/CD complexes to protons is hampered. The ratiometric titration plots (A_neutral_/A_cation_ vs. volume of the titration agent) (Figure 6B and insets in Figure 6C,D) show that the protonation of free camptothecin is complete after addition of 30 μL of HCl. In the case of CPT/CDs complexes, the addition of the same volume of acid produces absorbance ratios around 2 to 3, depending on the CD involved, showing protection towards protonation of camptothecin in the presence of cyclodextrins.

In the case of luotonin A, due to the presence of two basic nitrogens, leading to the absence of an isosbestic point, it is not easy to follow the overlapped proton transfer processes by UV-Vis absorption spectrophotometry [48]. As acid-base behavior is enhanced in the excited state, we chose to study the protection against protonation afforded by CD complexation by fluorimetry. Luotonin A exhibits a significant native fluorescence in different solvents, and acidification causes its protonation and an excited state proton transfer reaction (ESPT). Thus, the fluorescence emission of neutral and cationic forms are clearly different, with λ_ex_ = 342 nm, λ_em_ = 426 nm, for the neutral form and λ_ex_ = 392 nm, λ_em_ = 502 nm for the cationic form.

**Figure 7:**
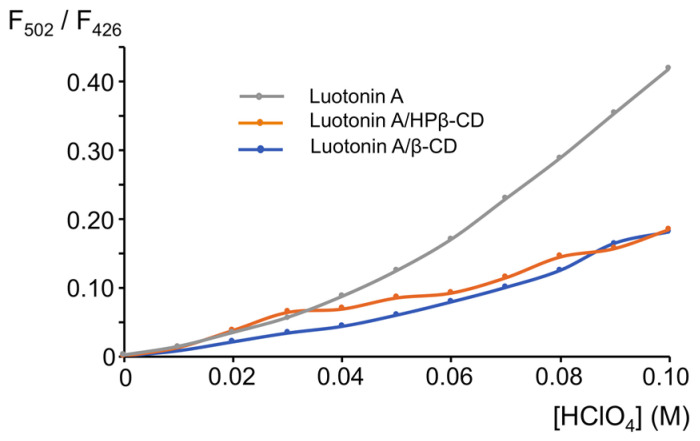
Ratiometric titration of luotonin A and its complexes with β-CD and HPβ-CD upon addition of increasing volumes (20 μL per addition) of 1.0 M HClO_4_.

The fluorescence emission intensities of luotonin A and its complexes with β-CD and HPβ-CD were processed in the course of their titration with perchloric acid. Solutions of native luotonin A as well as the inclusion complexes were prepared with the amount of alkaloid suitable to achieve a 1.0 × 10^−6^ M concentration in the aqueous solution. Increasing volumes of 1.0 M perchloric acid caused a decrease in the fluorescence emission of the neutral form (λ_ex_ = 342 nm, λ_em_ = 426 nm) accompanied by an increase of the emission intensity for the cationic form (λ_ex_ = 392 nm, λ_em_ = 502 nm). Because the absolute values of the fluorescence intensity for neutral and cationic forms are quantitatively different as a consequence of their different fluorescence quantum yields [49], we performed a ratiometric titration of the alkaloid and its complexes. As can be observed in Figure 7, protonation of luotonin A is notably easier for the free alkaloid than for the corresponding inclusion complexes. A final 0.1 M concentration of perchloric acid causes the protonation of luotonin A and a fluorescence intensity ratio cationic/neutral of 0.45 is reached. In the case of luotonin A-CD inclusion complexes, the cationic/neutral fluorescence intensity ratio was only 0.15. This result clearly shows that the protonation is hampered in the case of the CD complexes.

### 3.5. HPLC-FL Evaluation of the Stability of Camptothecin Inclusion Complexes

**Figure 8:**
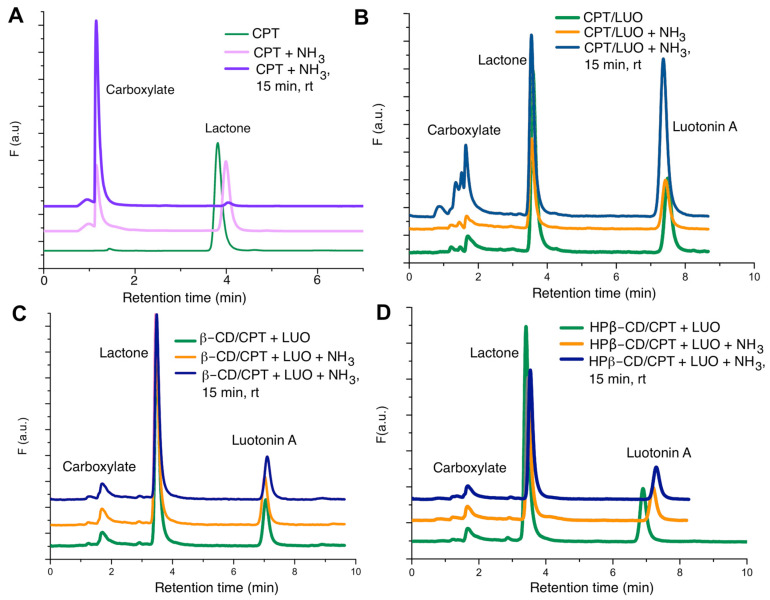
Chromatograms showing that CD complexation protects camptothecin from hydrolysis. (**A**) Hydrolysis of the camptothecin lactone E ring in the presence of aqueous ammonia. (**B**) The same reaction followed by separation in the presence of luotonin A as an internal standard. (**C**) Campothecin hydrolysis in the presence of β-cyclodextrin. (**D**) Campothecin hydrolysis in the presence of HP-β-CD.

In order to study the potential benefits of CPT-CD inclusion in terms of increased drug stability, a quantitative determination of the lactone form of camptothecin was performed by reverse phase HPLC with fluorescence detection (Figure 8).

We first determined the retention times of camptothecin in its active lactone form and the ring E-opened carboxy form derived from its hydrolysis (Figure 8A), using luotonin A as an internal standard for quantitative purposes (Figure 8B). Calibration curves for camptothecin and luotonin A mere obtained, with luotonin A at a fixed concentration (5.0 × 10^−9^ M) and camptothecin concentrations ranging from 1.0 × 10^−10^ M to 1.0 × 10^−8^ M. Linear regression parameters were obtained by plotting CPT area under the curve/luotonin A area under the curve against the CPT concentrations (Table S4). These calibration curves were employed to quantitate the concentrations of camptothecin lactone and consequently to establish the enhancement in the stability of camptothecin inclusion complexes. Figure 8B–D show the chromatograms obtained for the free alkaloid and the inclusion complexes with β-CD and HP-β-CD for the solutions prepared in MeCN: buffered aqueous solution (0.15 M ammonium acetate, pH = 6.0). Figure 8B shows the peak corresponding to camptothecin followed by the internal standard. At t = 0 (green chromatogram), the peak area of camptothecin is around 3–4 times higher than that of the internal standard, but the increase in pH and temperature causes the transformation of CPT lactone into CPT carboxylate, and thus the peak areas of lactone camptothecin and luotonin A become similar. In the case of the inclusion complexes (Figure 8C,D), the lactone camptothecin peak area is considerably higher than luotonin A peak area in the starting solution and remains with only a slight transformation into carboxylate camptothecin after 15 min under alkaline media at room temperature, leading to the conclusion that the alkaloid is protected from hydrolysis and subsequent degradation in the presence of CDs.

The quantitative values of degradation show that solutions of pure CPT lactone are transformed into carboxylate CPT, which constitutes around 40% of the degradation product under alkaline conditions at room temperature for 15 min. On the other hand, for the inclusion complexes with both β-CD and HP-β-CD, only a 3–7% of carboxylate form is generated under the same conditions. These results are in agreement with previous studies evidencing that CDs protect included compounds against different degradation reactions, mainly hydrolysis and oxidation reactions of pharmaceutically interesting products [47]. The quantitative data corresponding to the chemical degradation experiments are shown in the left half of Table 2.

Another series of experiments that revealed the stabilization of camptothecin associated to its inclusion in cyclodextrins were carried out in the presence of human serum albumin (HSA). As mentioned above, reversible hydrolysis of the camptothecin lactone leads to the inactive and nephrotoxic carboxylate species. This E ring opening is particularly problematic in therapeutic settings because the carboxylate form has a very high affinity (200-fold higher than the ring-closed lactone) for HSA, resulting in resulting in displacement of the equilibrium and a very high predominance of the inactive form [49,50]. For this reason, we studied the influence of CD complexation on the stability of camptothecin, finding that the complexes were 3–3.5 fold more stable than the free alkaloid towards E ring opening (right half of Table 2), which is a relevant finding in terms of the potential therapeutic use of the CD complexes.

### 3.6. Effect of Cyclodextrin Complexation on the Cytotoxicity of Camptothecin and Luotonin A

A crucial aspect of drug-cyclodextrin complexes is the comparison of the bioactivity of complexed and non-complexed compounds. For this reason, we studied the cytotoxicity of the free alkaloids and their complexes towards a series of cell lines representative of a variety of human solid tumors, namely AREc32 (breast cancer), H-23 (lung cancer), HepG2 (hepatic carcinoma), A2780 (ovarian carcinoma) and SH-SY5Y (neuroblastoma). The results are summarized in Table 3, expressed as CC_50_ values. As expected, camptothecin, a highly potent anticancer compound, showed cytotoxicities in the sub-micromolar range for all cell lines (entry 1). Gratifyingly, both the β-CD and the HP-β-CD complexes showed better activities than the reference alkaloid, ranging from ca. 2-fold to 7-fold higher potencies depending on the cell lines. Some of the measured cytotoxic activities are very high, being in the single-digit nanomolar range, namely the CPT/HP-β-CD complex against the HepG2 hepatic cancer cell line and the CPT/β-CD complex against the SH-SY5Y neuroblastoma cell line. In agreement with the literature, luotonin A was at least two orders of magnitude less potent than camptothecin (entry 4). Again, the cyclodextrin complexes improved the activity of the natural product up to ca. 80-fold, and in this case the HP-β-CD complexes were consistently more potent than their β-CD counterparts. These very significative increases in potency can be ascribed to improved solubilities, which are more relevant in the case of the highly lipophilic luotonin A. In the case of camptothecin, the increased stability must also contribute to the enhanced activity. It is relevant to note that luotonin A, which can be regarded as a stable natural analogue of camptothecin acting also as a topoisomerase I inhibitor, has not received much attention as an anticancer agent due to its relatively low potency. However, our results show that its HP-β-CD complex is a promising candidate that shows an activity comparable to that of camptothecin in some of the cell lines assayed, and in particular in the AREc32 (breast cancer) and H-23 (lung cancer) lines.

## 4. Conclusions

Camptothecin and luotonin A efficiently form inclusion complexes with β-CD and HP-β-CD, as demonstrated by NMR spectrometric techniques. These NMR data, together with docking and molecular dynamics computational studies, reveal that the structure of these complexes involves the full insertion of the camptothecin A and B rings in the cyclodextrin cavity, while luotonin A seems to be able to enter the cavity either by its A-B or D-E rings. Complementary spectroscopic assays show not only the inclusion into the cyclodextrin cavities but also the effective protection of the alkaloids against proton transfer reactions as well as the base-promoted hydrolysis of lactone ring in the case of camptothecin complexes, as quantified by HPLC. Interestingly from the point of view of potential therapeutic applications, CD complexation also protects camptothecin against serum albumin-induced lactone opening. The CD complexes showed enhanced cytotoxicity against a variety of human cancer cell lines with respect to the free alkaloids. In particular, the complex formed by luotonin A and HP-β-CD is more active than camptothecin in some cell lines and shows that the nanoencapsulation strategy may help to bring out the full potential of luotonin A as a stable analogue of camptothecin.

Taken in the aggregate, these findings underline how the use of β-CD and HP-β-CD as non-toxic drug delivery systems can improve the stability and solubility of the studied topoisomerase I inhibitors, eventually leading to an improved biological activity.

## Figures and Tables

**Table 1:** Association constants, given as *K*_ass_ (log *K*_ass_), obtained for the cyclodextrin complexes of camptothecin and luotonin A in aqueous solution at two pH values.

pH	CPT/β-CD	CPT/HP-β-CD	LUO A/β-CD	LUO A/HP-β-CD
5.5	668 (2.82)	227 (2.35)	103 (2.01)	242 (2.38)
1.0	65 (1.81)	84 (1.92)	11 (1.04)	91 (1.95)

**Table 2:** Quantitative data showing the stabilization of camptothecin following formation of its cyclodextrin complexes.

Compound	% Hydrolysis (aq. Ammonia, 15 min)	% Hydrolysis (Serum Albumin, 30 min)
Camptothecin (CPT)	40.2	4.98
CPT/β-CD complex	3.4	1.72
CPT/HPβ-CD complex	6.7	1.38

**Table 3:** Cytotoxicity induced in several cancer cell lines after 72 h incubation with camptothecin, luotonin A and their complexes with β-CD and HPβ-CD, expressed as CC_50_ (μM).

Entry	Compound	AREc32	H23	HepG2	A2780	SH-SY5Y
1	Camptothecin	0.489 ± 0.040	0.233 ± 0.030	0.066 ± 0.008	0.225 ± 0.074	0.023 ± 0.003
2	CPT/β-CD	0.222 ± 0.040	0.058 ± 0.014	0.013 ± 0.004	0.073 ± 0.041	0.009 ± 0.001
3	CPT/HPβ-CD	0.215 ± 0.029	0.030 ± 0.008	0.009 ± 0.002	0.106 ± 0.022	0.014 ± 0.002
4	Luotonin A	6.800 ± 0.440	31.010 ± 3.370	21.880 ± 7.700	45.570 ± 7.310	26.270 ± 6.500
5	Luo A/β-CD	0.589 ± 0.090	18.100 ± 1.770	0.796 ± 0.905	16.025 ± 2.290	7.800 ± 1.505
6	Luo A/HPβ-CD	0.139 ± 0.010	1.047 ± 0.550	0.283 ± 0.065	9.625 ± 1.770	4.620 ± 1.140

## Data Availability

The data presented in this study are available in the article and Supplementary Materials.

## Supplementary Materials

The following are available online at https://www.mdpi.com/article/10.3390/pharmaceutics13101609/s1, Figure S1: Fluorescence emission spectra of camptothecin in the presence of increasing concentrations of β-CD in aqueous solution at pH 5.5. Figure S2: Fluorescence emission spectra of camptothecin in the presence of increasing concentrations of HP-β-CD in aqueous solution at pH 5.5. Figure S3: Fluorescence emission spectra of luotonin A in the presence of increasing concentrations of β-CD in aqueous solution at pH 5.5. Figure S4: Fluorescence emission spectra of luotonin A in the presence of increasing concentrations of HP-β-CD in aqueous solution at pH 5.5. Figure S5: ^1^H-NMR spectrum of camptothecin (700 MHz, d_6_-DMSO). Figure S6. Overlapped ^1^H-NMR spectra of camptothecin (green) and its β-CD complex (red) (700 MHz, d_6_-DMSO). Figure S7. Overlapped ^1^H-NMR spectra of camptothecin (green) and its HP-β-CD complex (red) (700 MHz, d_6_-DMSO). Figure S8: Overlapped ^1^H-NMR spectra of luotonin A (red) and its β-CD complex (green) (700 MHz, d_6_-DMSO). Figure S9: Overlapped ^1^H-NMR spectra of luotonin A (red) and its HP-β-CD complex (green) (700 MHz, d_6_-DMSO). Figure S10: Comparison of the evolution of RMSD values for the camptothecin-β-CD 1:1 complex (purple), the luotonin A-β-CD 1:1 complex (blue) and the luotonin A-β-CD 1:2 complex (orange). Table S1: ^1^H-NMR chemical shift variations obtained for the inclusion complexes CPT/β-CD and CPT/HPβ-CD) with regard to those observed for the free drug in d_6_-DMSO. The variations correspond to the changes in the signals of the guest molecule. Table S2: ^13^C-NMR chemical shift variations obtained for the inclusion complexes CPT/β-CD and CPT/HPβ-CD) with regard to those observed for the free drug in d_6_-DMSO. Table S3: ^1^H-NMR chemical shift variations obtained for the inclusion complexes Luotonin A/β-CD and Luotonin A/HPβ-CD) with regard to those observed for the free drug in d_6_-DMSO. The variations correspond to the changes in the signals of the guest molecule. Table S4. Linear regression parameters obtained for the calibration curves of camptothecin and employing luotonin A as inner standard. The solutions of camptothecin and luotonin A were prepared in a 40:60; v:v mixture of acetonitrile: buffered aqueous solution 0.15 M (ammonium acetate/acetic acid) to obtain the desired pH value.

## References

[1] Hevener K.E., Verstak T.A., Lutat K.E., Riggsbee D.L., Mooney J.W. (2018). Recent developments in topoisomerase-targeted cancer chemotherapy. Acta Pharm. Sin. B..

[2] Avendaño C., Menéndez J.C. (2015). Medicinal Chemistry of Anticancer Drugs.

[3] Venditto V.J., Simanek E.E. (2010). Cancer therapies utilizing the camptothecins: A review of the in vivo literature. Mol. Pharm..

[4] Malgorzata N.D., Agama K., Wakelin L.P.G., Pommier Y., Griffith R. (2011). Exploring DNA topoisomerase I ligand space in search of novel anticancer agents. PLoS ONE.

[5] Li Q.-Y., Zu Y.-G., Shi R.-Z., Yao L.-P. (2006). Review camptothecin: Current perspectives. Curr. Med. Chem..

[6] Kacprzak K.M., Ramawat K., Mérillon J.M. (2013). Chemistry and biology of camptothecin and its derivatives. Natural Products.

[7] Thambi T., Yoon H.Y., Kim K., Kwon I.C., Yoo C.K., Park J.H. (2011). Bioreducible block copolymers based on poly(ethyleneglycol) and poly(γ-benzyl L-glutamate) for intracellular delivery of camptothecin. Bioconjug. Chem..

[8] Galbiati A., Tabolacci C., Della Rocca B.M., Mattioli P., Beninati S., Paradossi G., Desideri A. (2011). Targeting tumor cells through chitosan-folate modified microcapsules loaded with camptothecin. Bioconjug. Chem..

[9] Botella P., Abasolo I., Fernández Y., Muniesa C., Miranda S., Quesada M., Ruiz J., Schwartz S.J., Corma A. (2011). Surface-modified silica nanoparticles for tumor-targeted delivery of camptothecin and its biological evaluation. J. Controll. Release.

[10] Aydin A., Sipahi H., Charehsaz M., Sezer A.D. (2012). Nanoparticles toxicity and their routes of exposures. Recent Advances in Novel Drug Carrier Systems.

[11] Liang J.L., Cha H.C., Jahng Y. (2011). Recent advances in the studies on luotonins. Molecules.

[12] Cagir A., Eisenhauer B.M., Gao R., Thomas S.J., Hecht S.M. (2004). Synthesis and topoisomerase I inhibitory properties of luotonin A analogues. Bioorg. Med. Chem..

[13] Golubev A.S., Bogomolov V.O., Shidlovskii A.F., Dezhenkova L.G., Peregudov A.S., Shtil A.A., Chkanikov N.D. (2010). Synthesis of fluoromethyl-containing analogs of antitumor alkaloid luotonin A. Russ. Chem. Bull..

[14] González-Ruiz V., Pascua I., Fernández-Marcelo T., Ribelles P., Bianchini G., Sridharan V., Iniesta P., Ramos M.T., Olives A.I., Martín M.A. (2014). B-ring-aryl substituted luotonin A analogues with a new binding mode to the topoisomerase 1-DNA complex show enhanced cytotoxic activity. PLoS ONE.

[15] Almansour A.I., Arumugam N., Suresh Kumar R., Mahalingam S.M., Sau S., Bianchini G., Menéndez J.C., Altaf M., Ghabbour A. (2017). Design, synthesis and antiproliferative activity of decarbonyl luotonin analogues. Eur. J. Med. Chem..

[16] Almansour A.I., Suresh Kumar R., Arumugam N., Bianchini G., Menéndez J.C., Mohammad F., Dupadahalli K., Altaf M. (2019). D-ring-modified analogues of luotonin A with reduced planarity: Design, synthesis, and evaluation of their topoisomerase inhibition-associated cytotoxicity. BioMed Res. Int..

[17] Yang G.-Z., Zhang J., Peng J.-W., Zhang Z.-J., Zhao W.-B., Wang R.-X., Ma K.-Y., Li J.-C., Liu Y.-Q., Zhao Z.-M. (2020). Discovery of luotonin A analogues as potent fungicides and insecticides: Design 4524, synthesis and biological evaluation inspired by natural alkaloid. Eur. J. Med. Chem..

[18] Hao Y., Wang K., Wang Z., Liu Y., Ma D., Wang Q. (2020). Luotonin A and its derivatives as novel antiviral and antiphytopathogenic fungus agents. J. Agric. Food Chem..

[19] Uekama K., Hirayama F., Wermuth C.G. (2008). Improvement of drug properties by cyclodextrins. The practice of Medicinal Chemistry.

[20] Qiu N., Li X., Liu J. (2017). Application of cyclodextrins in cancer treatment. J. Incl. Phenom. Macrocycl. Chem..

[21] Gidwani B., Vyas A. (2015). A comprehensive review on cyclodextrin-based carriers for delivery of chemotherapeutic cytotoxic anticancer drugs. BioMed Res. Int..

[22] Saokham P., Muankaew C., Jansook P., Loftsson T. (2018). Solubility of cyclodextrins and drug/cyclodextrin complexes. Molecules.

[23] Popielec A., Loftsson T. (2017). Effects of cyclodextrins on the chemical stability of drugs. Int. J. Pharm..

[24] Diniz T.C., Pinto T.C.C., Menezes P.P., Silva J.C., Teles R.B.A., Ximenes R.C.C., Guimarães A.G., Serafini M.R., Araújo A.A.S., Quintans L.J. (2018). Cyclodextrins improving the physicochemical and pharmacological properties of antidepressant drugs: A patent review. Expert Opin. Ther. Pat..

[25] Li X., Uehara S., Sawangrat K., Morishita M., Kusamori K., Katsumi H., Sakane T., Yamamoto A. (2018). Improvement of intestinal absorption of curcumin by cyclodextrins and the mechanisms underlying absorption enhancement. Int. J. Pharm..

[26] Popielec A., Agnes M., Yannakopoulou K., Fenyvesi É., Loftsson T. (2018). Effect of β- and γ-cyclodextrins and their methylated derivatives on the degradation rate of benzylpenicillin. J. Incl. Phenom. Macrocycl. Chem..

[27] Malaquias L.F.B., Sá-Barreto L.C.L., Freire D.O., Silva I.C.R., Karan K., Durig T., Lima E.M., Marreto R.N., Gelfuso G.M., Gratieri T. (2018). Taste masking and rheology improvement of drug complexed with beta-cyclodextrin and hydroxypropyl-β-cyclodextrin by hot-melt extrusion. Carbohydr. Polym..

[28] Chattah A.K., Pfund L.Y., Zoppi A., Longhi M.R., Garnero C. (2017). Toward novel antiparasitic formulations: Complexes of albendazole desmotropes and β-cyclodextrin. Carbohydr. Polym..

[29] Michalska P., Wojnicz A., Ruiz-Nuño A., Abril S., Buendia I., León R. (2017). Inclusion complex of ITH12674 with 2-hydroxypropyl-β-cyclodextrin: Preparation, physical characterization and pharmacological effect. Carbohydr. Polym..

[30] Yee E.M.H., Hook J.M., Bhadbhade M.M., Vittorio O., Kuchel R.P., Brandl M.B., Tilley R.D., Black St C.D., Kumar N. (2017). Preparation, characterization and in vitro biological evaluation of (1:2) phenoxodiol-β-cyclodextrin complex. Carbohydr. Polym..

[31] Braga Carneiro S., Costa Duarte F.Í., Heimfarth L., de Souza Siqueira Quintans J., Quintans-Júnior L.J., Florêncio da Veiga Júnior V., Neves de Lima A.A. (2019). Cyclodextrin–drug inclusion complexes: In vivo and in vitro approaches. Int. J. Mol. Sci..

[32] Rodríguez-Cáceres M.I., Bohoyo D., Durán-Merás I., Hurtado M.C. (2011). Spectrofluorimetric determination of SN-38, a promising new anti-tumor agent, in the presence and absence of organized media. Appl. Spectrosc..

[33] Foulon C., Tedou J., Queruau Lamerie T., Vaccher C., Bonte J.P., Goossens J.F. (2009). Assessment of the complexation degree of camptothecin derivatives and cyclodextrins using spectroscopic and separative methodologies. Tetrahedron Asymmetry.

[34] Kang J., Kumar V., Yang D., Chowdhury P.R., Hohl R.J. (2002). Cyclodextrin complexation: Influence on the solubility, stability, and cytotoxicity of camptothecin, an antineoplastic agent. Eur. J. Pharm. Sci..

[35] Sætern A.M., Nguyen N.B., Bauer-Brandl A., Brandl M. (2004). Effect of hydroxypropyl- β-cyclodextrin complexation and pH on solubility of camptothecin. Int. J. Pharm..

[36] Cheng J.G., Yu H.-J., Chen Y., Liu Y. (2018). Selective binding and controlled release of anticancer drugs by polyanionic cyclodextrins. Bioorg. Med. Chem..

[37] Jiang Y., Sha X., Zhang W., Fang X. (2010). Complex of 9-nitro-camptothecin in hydroxypropyl-β-cyclodextrin: In vitro and in vivo evaluation. Int. J. Pharm..

[38] Jiang Y., Jiang X., Law K., Chen Y., Gu J., Zhang W., Xin H., Sha X., Fang X. (2011). Enhanced anti-tumor effect of 9-nitro-camptothecin complexed by hydroxypropyl-β-cyclodextrin and safety evaluation. Int. J. Pharm..

[39] Ünal H., Öztürk N., Bilensoy E. (2015). Formulation development, stability and anticancer efficacy of core-shell cyclodextrin nanocapsules for oral chemotherapy with camptothecin. Beilstein J. Org. Chem..

[40] Çirpanli Y., Bilensoy E., Doğan A.L., Çaliș S. (2009). Comparative evaluation of polymeric and amphiphilic cyclodextrin nanoparticles for effective camptothecin delivery. Eur. J. Pharm. Biopharm..

[41] González-Ruiz V., Olives A.I., Martín M.A. (2013). A down-scaled fluorimetric determination of the solubility properties of drugs to minimize waste generation. Green Chem..

[42] Di Nunzio M.R., Cohen B., Douhal A. (2011). Structural photodynamics of camptothecin, an anticancer drug in aqueous solutions. J. Phys. Chem. A.

[43] González-Ruiz V., Mussardo P., Corda E., Girotti S., Olives A.I., Martín M.A. (2010). Liquid chromatographic analysis of the anticancer alkaloid luotonin A and some new derivatives in human serum samples. J. Sep. Sci..

[44] Day J., Warner I.M. (1996). Exceited state tautomerization of camptothecin in aqueous solution. J. Photochem. Photobiol. A Chem..

[45] Schalley C. (2012). . Analytical Methods In Supramolecular Chemistry.

[46] Connors K.A., Szejtli J., Osa T. (1996). Measurement of cyclodextrin complex stability constants. Cyclodextrins.

[47] Martín del Valle E.M. (2004). Cyclodextrins and their uses: A review. Process Biochem..

[48] González-Ruiz V., González-Cuevas Y., Arunachalam S., Martín M.A., Olives A.I., Ribelles P., Ramos M.T., Menéndez J.C. (2012). Fluorescence properties of the anti-tumour alkaloid luotonin A and new synthetic analogues: pH modulation as an approach to their fluorimetric quantitation in biological samples. J. Lumin..

[49] Mi Z., Burke T.G. (1994). Differential interactions of camptothecine lactone and carboxylate forms with human blood components. Biochemistry.

[50] Pistolozzi M., Varchi G., Degli Esposti A., Guerrini A., Sotgiu G., Ballestri M., Ferroni C., Venturini A., Bertucci C. (2011). Camptothecin and thiocamptothecin: The role of sulfur in shifting the hydrolysis equilibrium towards the closed lactone form. ChemMedChem.

