# Supplementary material for: Enhanced Stability and Bioactivity of Natural Anticancer Topoisomerase I Inhibitors through Cyclodextrin Complexation
Source: Pharmaceutics. 2021 Oct 3;13(10):1609. doi: 10.3390/pharmaceutics13101609 (PMC8537677; doi:10.3390/pharmaceutics13101609)
Supplement: Supplementary file 1 [file pharmaceutics-13-01609-s001.zip › pharmaceutics-1392826-SM.pdf]

pharmaceutics

MDPI

Supplementary Materials: Enhanced stability and bioactivity of natural anticancer topoisomerase I inhibitors through cyclodextrin nanoencapsulation

Víctor González-Ruiz, Ángel Cores, Olmo Martín-Cámara, Karen Orellana, Víctor Cervera- Carrascón, Patrycja Michalska, Ana I. Olives, Rafael León, M. Antonia Martín and J. Carlos Menéndez

Table S1. ^{1}H-NMR chemical shift variations obtained for the inclusion complexes CPT/β-CD and CPT/HPβ-CD) with regard to those observed for the free drug in d_6- DMSO. The variations correspond to the changes in the signals of the guest molecule.

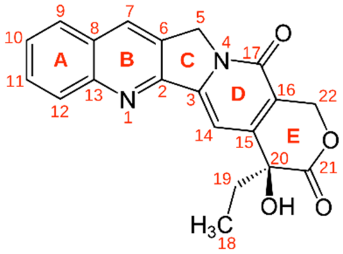

.

H-SIGNAL	CPT/β-CD complex		CPT/HPβ-CD complex	
	Δδ (absolute, ppm)	Δδ (normalized, ppm)	Δδ (absolute, ppm)	Δδ (normalized, ppm)
H-5	0.017	1.0	0.021	1.0
H-7	0.015	0.882	0.018	0.857
H-9	0.014	0.823	0.017	0.809
H-10	0.010	0.588	0.013	0.619
H-11	0.009	0.529	0.012	0.571
H-12	0.011	0.647	0.014	0.667
H-14	0.007	0.412	0.010	0.476
H-18	0.000	0.000	0.000	0.000
H-19	0.001	0.059	0.003	0.143
H-22	0.005	0.294	0.006	0.286

Table S2. 13C-NMR chemical shift variations obtained for the inclusion complexes CPT/β-CD and CPT/HPβ-CD) with regard to those observed for the free drug in d6- DMSO.

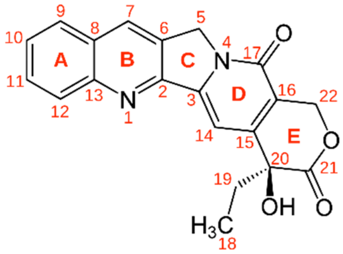

C-SIGNAL	CPT/β-CD complex		CPT/HPβ-CD complex	
	Δδ (absolute, ppm)	Δδ (normalized, ppm)	Δδ (absolute, ppm)	Δδ (normalized, ppm)
C-2	0.035	0.921	0.040	1.0
C-3	0.023	0.605	0.027	0.675
C-5	0.020	0.526	0.027	0.675
C-6	0.020	0.526	0.022	0.550
C-7	0.013	0.342	0.016	0.400
C-8	0.018	0.474	0.025	0.625
C-9	0.026	0.684	0.031	0.775
C-10	0.015	0.395	0.023	0.575
C-11	0.022	0.579	0.027	0.675
C-12	0.038	1.0	0.023	0.575
C-13	0.019	0.500	0.020	0.500
C-14	0.008	0.210	--	--
C-15	0.008	0.210	0.008	0.200
C-16	0.015	0.395	0.016	0.400
C-17	0.013	0.342	0.019	0.475
C-18	0.001	0.026	--	--
C-19	0.007	0.184	0.007	0.175
C-20	0.008	0.210	--	--
C-21	--	--	--	--
C-22	0.003	0.079	0.004	0.100

Table S3. ^{1}H-NMR chemical shift variations obtained for the inclusion complexes Luotonin A/β-CD and Luotonin A /HPβ-CD) with regard to those observed for the free drug in d_6-DMSO. The variations correspond to the changes in the signals of the guest molecule.

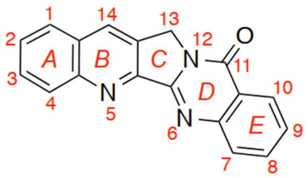

H-SIGNAL	Luotonin A/β-CD complex Δδ (absolute, ppm),	Δδ (normalized, ppm)	Luotonin A/HPβ-CD complex Δδ (absolute, ppm)	Δδ (normalized, ppm)
H-1	0.004	1.0	0.004	1.0
H-2	0.003	0.750	0.003	0.750
H-3	0.003	0.750	0.003	0.750
H-4	0.003	0.750	0.003	0.750
H-5	--	--	--	--
H-6	--	--	--	--
H-7	0.003	0.750	0.003	0.750
H-8	0.003	0.750	0.003	0.750
H-9	0.003	0.750	0.003	0.750
H-10	0.004	1.0	0.004	1.0
H-11	--	--	--	--
H-12	--	--	--	--
H-13	0.004	1.0	0.004	1.0
H-14	0.004	1.0	0.004	1.0

Table S4. Linear regression parameters obtained for the calibration curves of CPT and employing luotonin A as inner standard. The solutions of CPT and luotonin A were prepared in a 40:60; v:v mixture of acetonitrile : buffered aqueous solution 0.15 M (ammonium acetate/acetic acid) to obtain the desired pH value.

pH	Linear range studied	R^2	Ordinate	Slope
3.0	5.0×10^{-10} to 1.0×10^{-8} M	0.9865	0.61	1.0×10^{9}
6.0	5.0×10^{-10} to 1.0×10^{-8} M	0.9923	0.25	2.0×10^{9}
8.0	5.0×10^{-10} to 1.0×10^{-8} M	0.9833	1.95	9.0×10^{8}

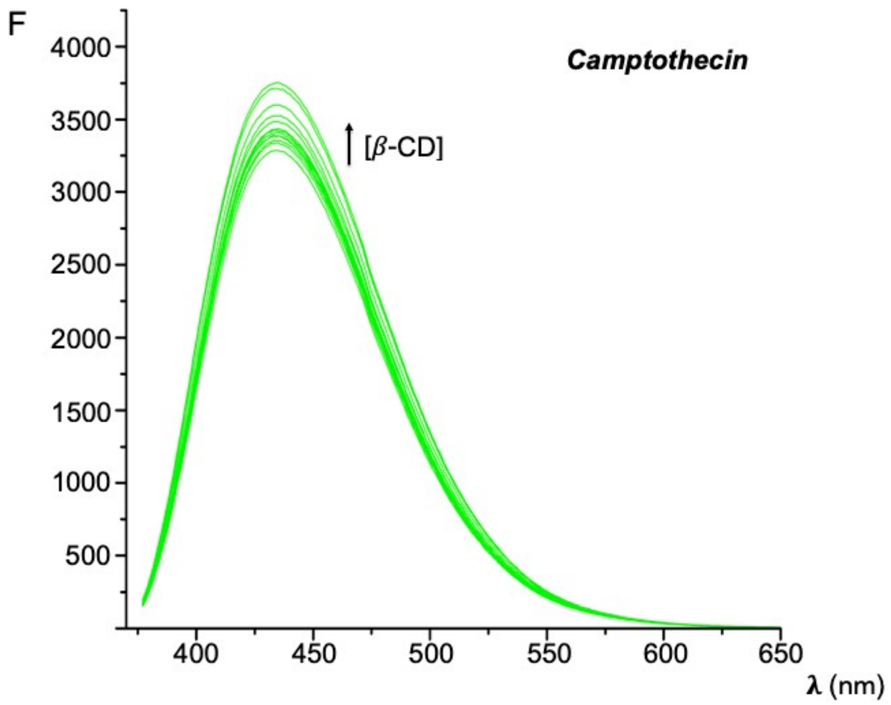

Figure S1. Fluorescence emission spectra of camptothecin in the presence of increasing concentrations of β-CD in aqueous solution at pH 5.5.

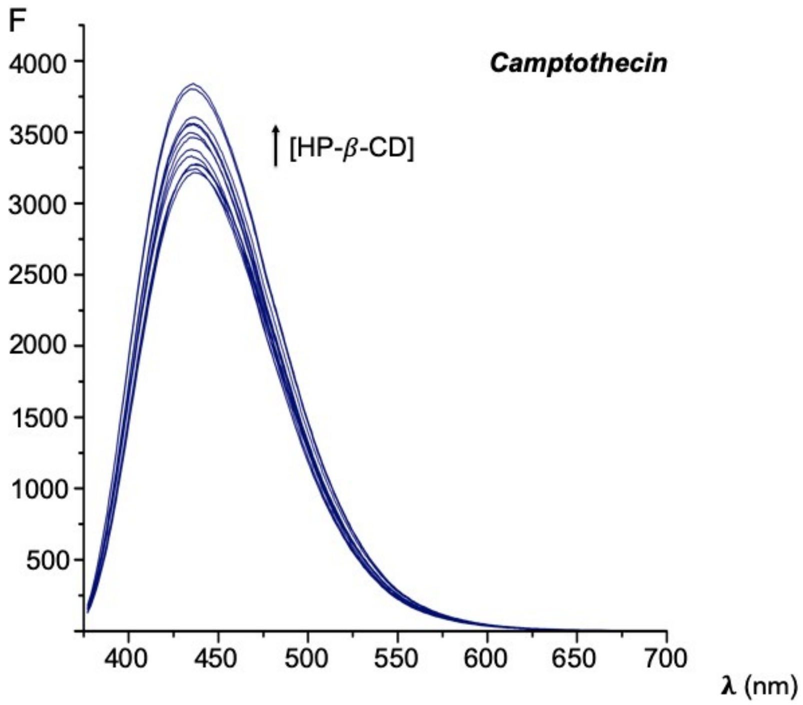

Figure S2. Fluorescence emission spectra of camptothecin in the presence of increasing concentrations of HP-β-CD in aqueous solution at pH 5.5.

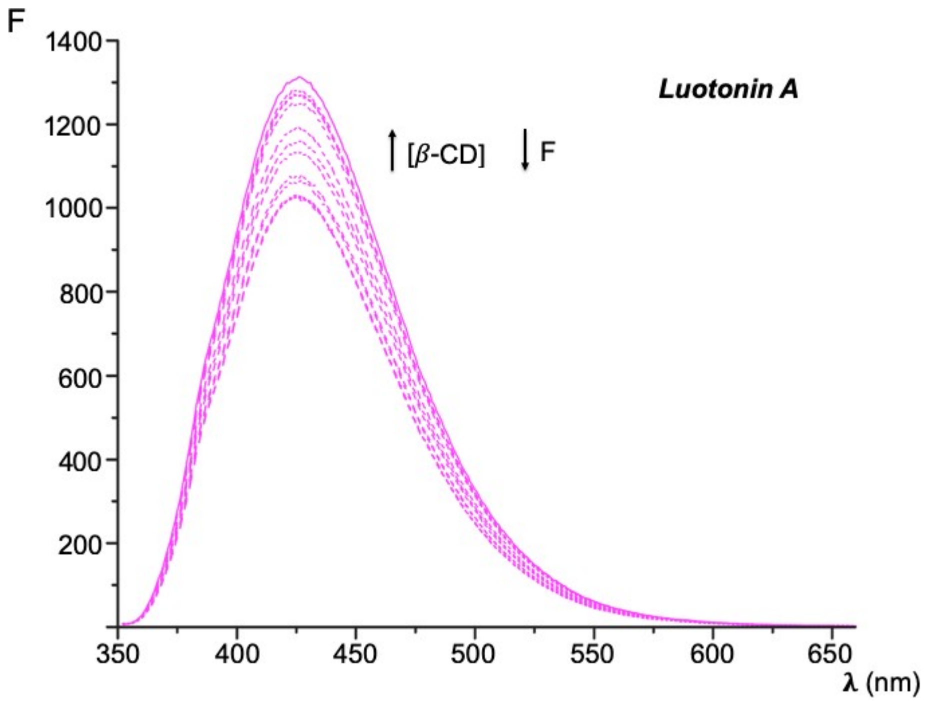

Figure S3. Fluorescence emission spectra of luotonin A in the presence of increasing concentrations of β-CD in aqueous solution at pH 5.5.

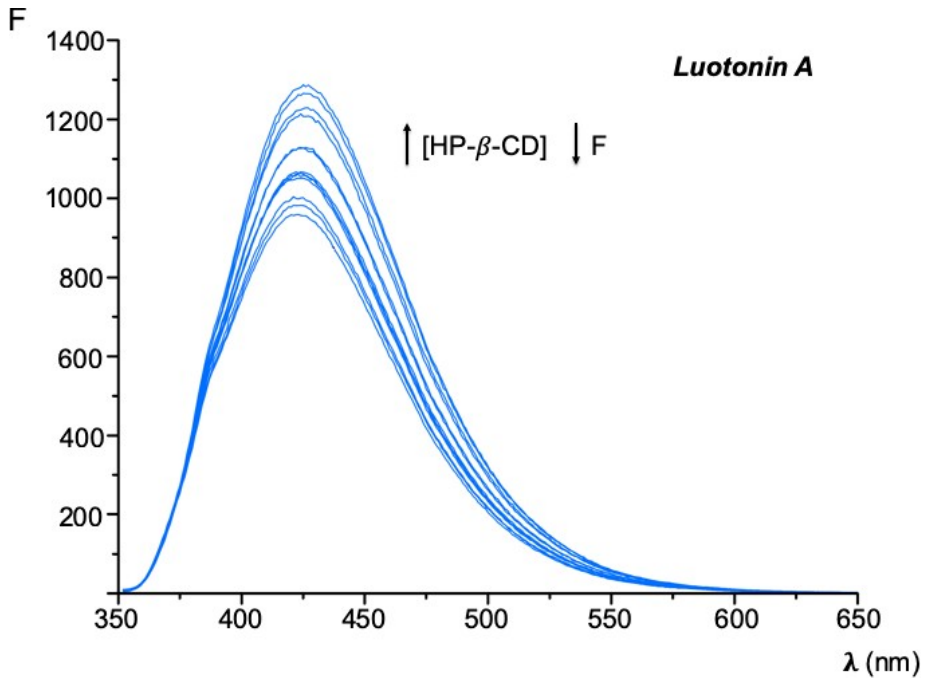

Figure S4. Fluorescence emission spectra of luotonin A in the presence of increasing concentrations of HP-β-CD in aqueous solution at pH 5.5.

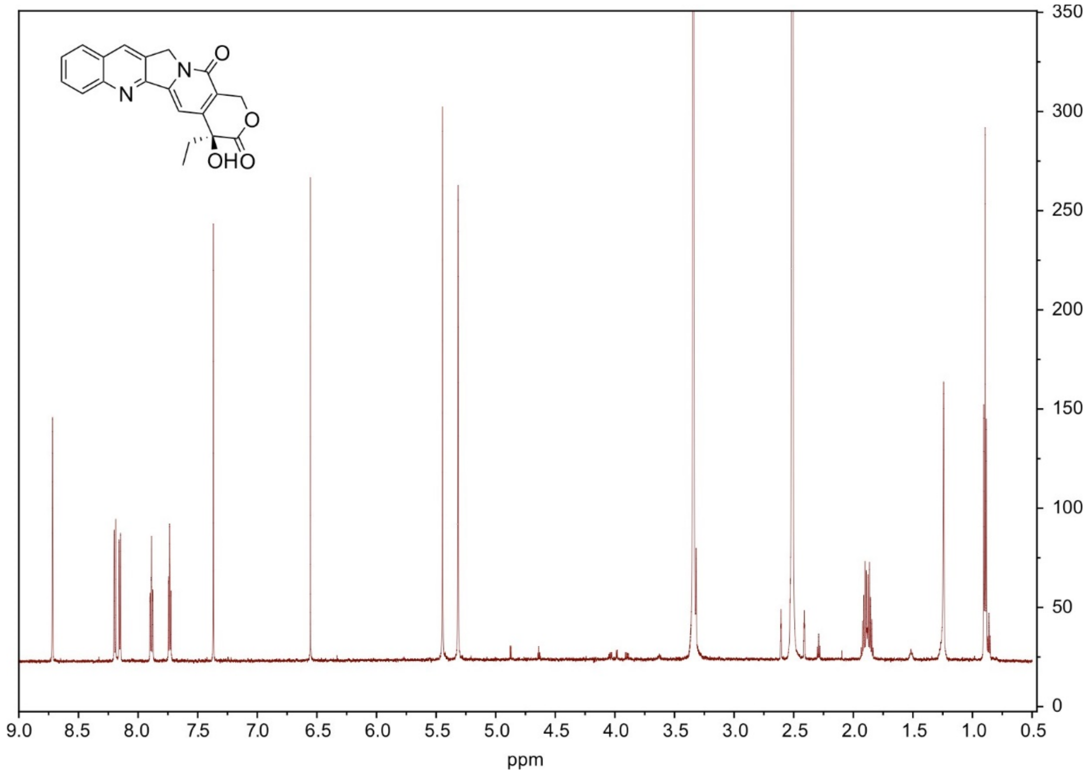

Figure S5. ^{1}H-NMR spectrum of camptothecin (700 MHz, d_6-DMSO).

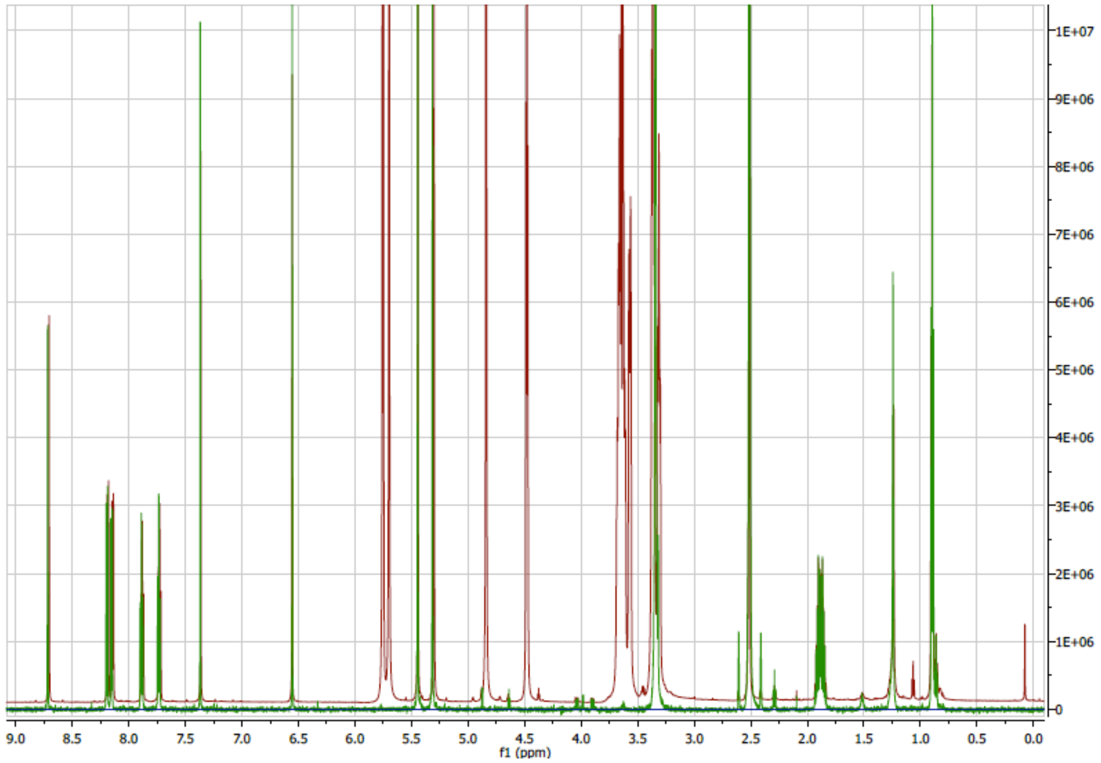

Figure S6. Overlapped ^{1}H-NMR spectra of camptothecin (green) and its β-CD complex (red) (700 MHz, d_6-DMSO).

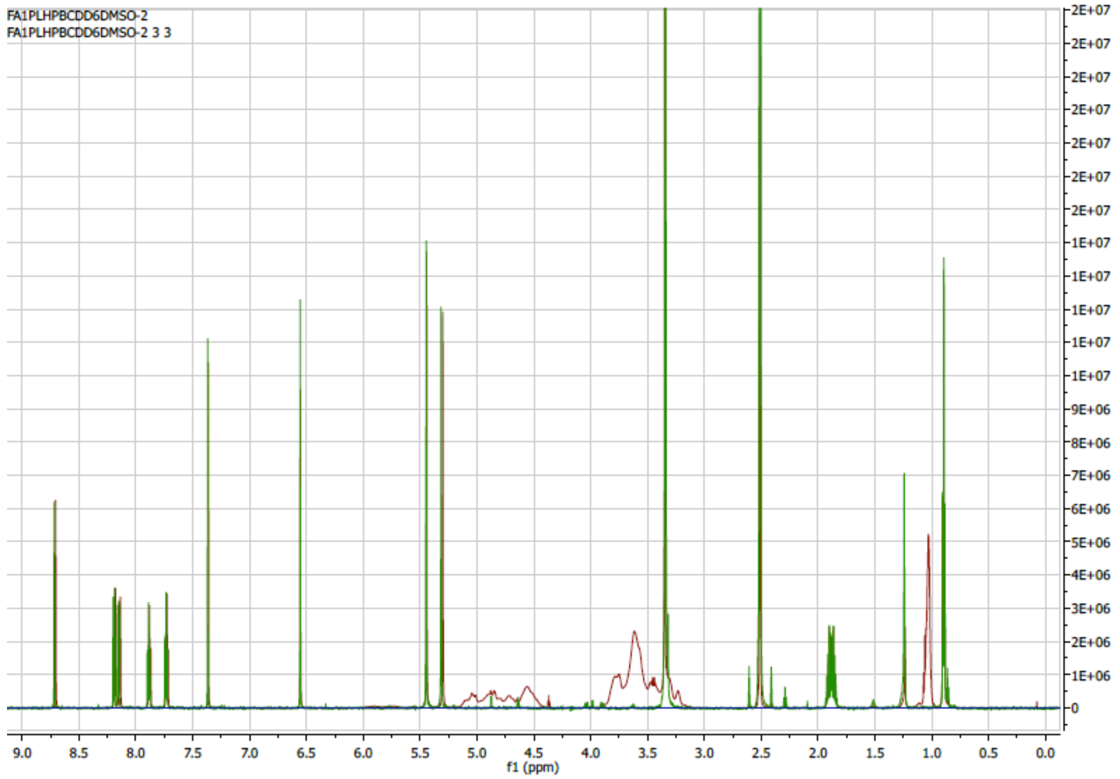

Figure S7. Overlapped ^{1}H-NMR spectra of camptothecin (green) and its HP-β-CD complex (red) (700 MHz, d_6-DMSO).

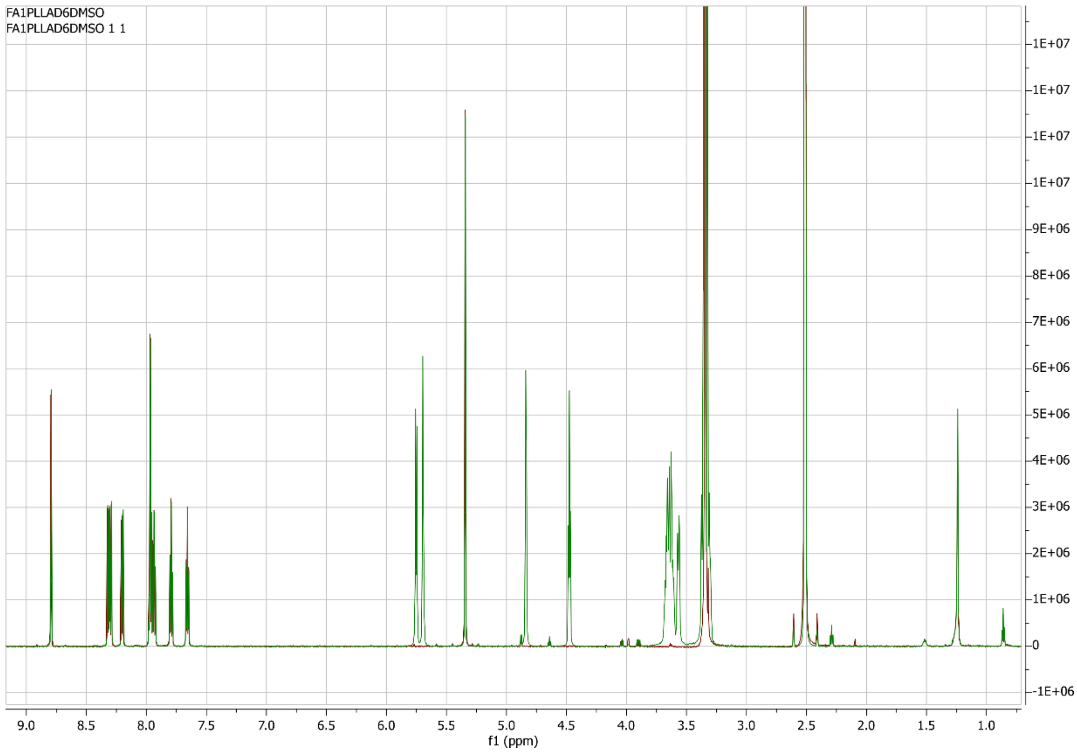

Figure S8. Overlapped ^{1}H-NMR spectra of luotonin A (red) and its β-CD complex (green) (700 MHz, d_6-DMSO).

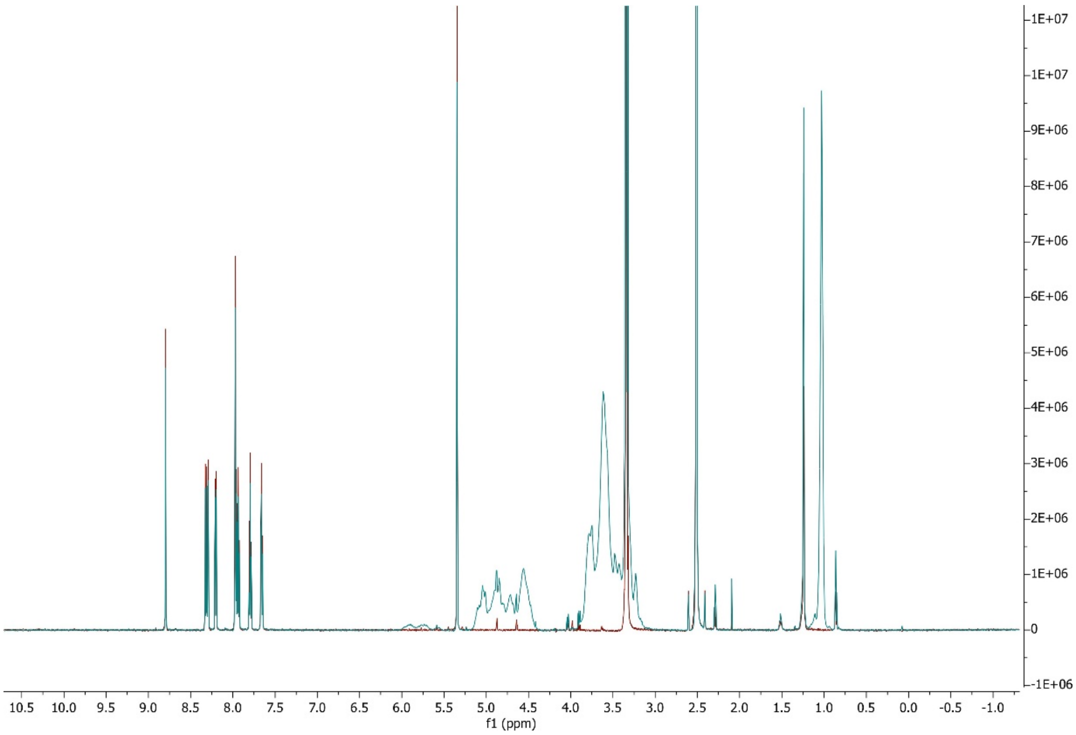

Figure S9. Overlapped ^{1}H-NMR spectra of luotonin A (red) and its HP-β-CD complex (green) (700 MHz, d_6-DMSO).

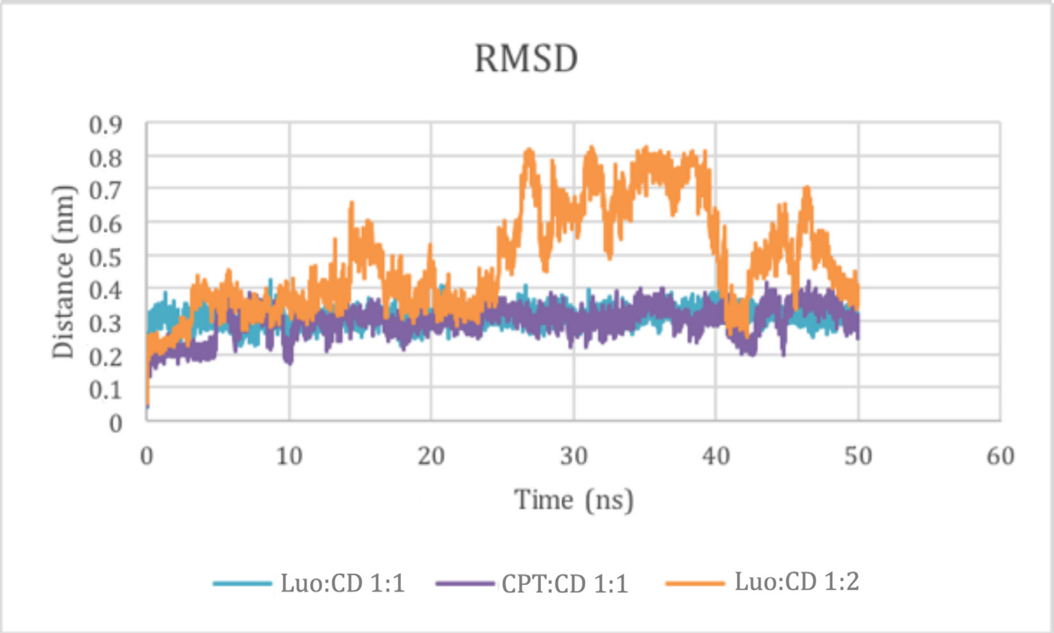

Figure S10. RMSD values for the camptothecin-β-CD 1:1 complex (purple), the luotonin-β-CD 1:1 complex (blue) and the luotonin-β-CD 1:2 complex (orange).
